# MicroRNA-214 Mediates Isoproterenol-induced Proliferation and Collagen Synthesis in Cardiac Fibroblasts

**DOI:** 10.1038/srep18351

**Published:** 2015-12-22

**Authors:** Min Sun, Haiyi Yu, Youyi Zhang, Zijian Li, Wei Gao

**Affiliations:** 1 Department of Cardiology, Peking University Third Hospital and Key Laboratory of Cardiovascular Molecular Biology and Regulatory Peptides, Ministry of Health, Key Laboratory of Molecular Cardiovascular Sciences, Ministry of Education and Beijing Key Laboratory of Cardiovascular Receptors Research Beijing 100191, China

## Abstract

The action of β-adrenergic receptors (β-ARs) induces cardiac fibroblast (CF) proliferation and collagen synthesis and is a major source of the cardiac fibrosis caused by various diseases. Recently, microRNA-214 (miR-214) was found to play an important role in the pathogenesis of cardiac remodelling. In the present study, we examined the role and the underlying mechanism of miR-214 in isoproterenol (ISO, a β-AR agonist)-induced CF proliferation and collagen synthesis. The expression of miR-214 was increased in both ISO-mediated fibrotic heart tissue and fibroblasts. Downregulation of miR-214 by antagonists attenuated the proliferation and collagen synthesis in ISO-treated CFs. Using bioinformatics analysis and luciferase assays, mitofusin2 (Mfn2), a critical regulator of cell proliferation and tissue fibrosis, was identified as a direct target gene of miR-214; this result was confirmed by western blot analysis. Additionally, corresponding to the upregulation of miR-214, the expression of Mfn2 was downregulated in the fibrotic heart and fibroblasts. Furthermore, the downregulation of miR-214 inhibited the activation of ERK1/2 MAPK signalling induced by ISO treatment. In conclusion, our study demonstrated that miR-214 mediates CF proliferation and collagen synthesis via inhibition of Mfn2 and activation of ERK1/2 MAPK signalling, which provides a new explanation for the mechanism of β-AR activation-induced cardiac fibrosis.

Cardiac fibrosis is an important pathological change occurring in cardiac remodelling following ischemic heart disease, hypertension, cardiomyopathy, and other diseases. This phenomenon contributes to the impairment of pump function and provides the basis for heart failure^1^. The proliferation of cardiac fibroblasts (CFs) and excessive deposition of extracellular matrix (ECM) proteins such as collagen types I and III are the major characteristics of cardiac fibrosis^2^. β-adrenergic receptors (β-ARs), the dominant adrenergic receptors in the heart, have been reported to be excessively stimulated in various cardiovascular diseases^3^ and play a critical role in cardiac fibrosis^4^. Excessive β-AR stimulation can promote the proliferation and collagen synthesis of cardiac fibroblasts by activating ERK1/2 MAPK and p38 MAPK signalling, transactivating the epidermal growth factor receptor, and inducing the production of cytokines^4^,^5^,^6^,^7^. However, the function and mechanism of microRNAs in β-AR-mediated cardiac fibrosis remain unclear.

miRNAs are 18- to 25-nucleotide conserved noncoding RNAs that negatively regulate gene expression through mRNA cleavage or translational repression by base pairing with complementary sequences in the 3′ untranslated regions (3′UTRs) of target genes. Recent studies have revealed that miRNAs play an important role in the pathogenesis of cardiac fibrosis^8^. Cardiac-specific deletion of the endonuclease Dicer, which is required for miRNA maturation, has been shown to result in cardiac hypertrophy and myocardial fibrosis^9^. Several miRNAs, including miR-133a^10^,^11^, miR-206^12^, miR-21^13^,^14^ and miR-29b^15^,^16^, have been reported to actively participate in cardiac fibrosis by controlling collagen synthesis and degradation, fibroblast proliferation, and the key signalling pathways regulating fibrosis. Moreover, a recent study demonstrated that β-ARs can regulate miRNA expression in the rat heart^17^, suggesting that miRNAs may mediate β-AR-induced cardiac fibrosis.

miR-214, a sensitive marker of cardiac stress, was found to be upregulated in hearts overactivated by β-ARs, using a miRNA array test^17^. This upregulation can provoke cardiac hypertrophy and heart failure^18^. In addition, recent studies have also reported that downregulation of miR-214 can attenuate unilateral ureteral obstruction (UUO)-induced renal fibrosis^19^. These studies suggest that miR-214 may play an important role in cardiac fibrosis induced by excessive stimulation of β-ARs.

In the present study, we explored the role and mechanism of miR-214 in isoproterenol (ISO, a β-AR agonist)-induced cardiac fibrosis. Our results show that miR-214 mediates ISO-induced proliferation and collagen synthesis in CFs by directly targeting Mfn2 and activating the downstream extracellular signal-regulated kinase–mitogen-activated protein kinase (ERK1/2 MAPK) signalling pathway.

## Results

### miR-214 is upregulated in ISO-induced cardiac fibrosis

**Figure 1:**
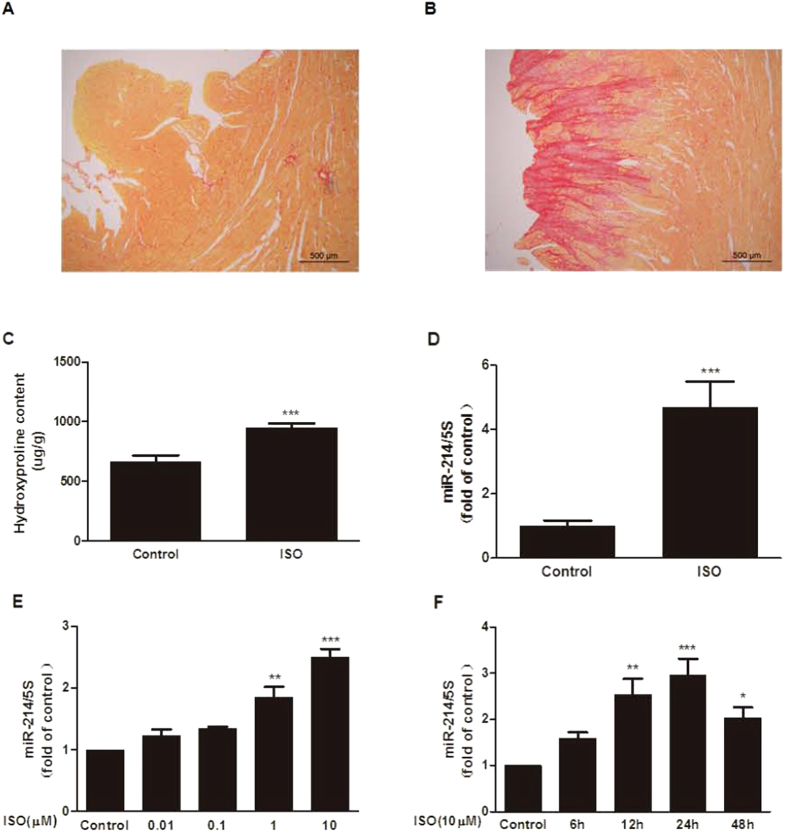
miR-214 is upregulated in ISO-induced fibrotic heart tissue and CFs. (**A**) Histopathological features of collagen deposition by Sirius red staining of heart sections from ISO-treated rats (100× magnification). (**B**) ISO enhanced the hydroxyproline content in cardiac tissue (n = 7 for each group, mean ± SEM, ^***^P < 0.001 *vs*. Control). (**C**) The expression of miR-214 was increased in the myocardium of rats treated with ISO for 7 days compared to the control (n = 7 for each group, mean ± SEM, ^***^P < 0.001 *vs*. Control). (**D**) CFs were incubated with various concentrations of ISO for 24 h, and miR-214 levels were measured by quantitative real-time PCR. (**E**) CFs were treated with 10 μM ISO at the indicated time points (n = 5, mean ± SEM, ^*^P < 0.05, ^**^P < 0.001, ^***^P < 0.001 *vs.* Control).

Previous studies have demonstrated that the expression of miR-214 is upregulated in the ISO-treated rat heart^17^; thus, we first examined whether the level of miR-214 also changes in an ISO-induced cardiac fibrosis model. *In vivo*, SD rats were treated with subcutaneous injection of ISO (0.25 mg/kg) once a day for 7 consecutive days, and histological staining with Sirius red and hydroxyproline quantification were then performed to evaluate cardiac fibrosis. Compared with the control group, the cardiac interstitial fibrosis area and collagen content increased following ISO injection (Fig. 1A,B), demonstrating that the cardiac fibrosis model was successfully established. Furthermore, the level of miR-214 was significantly increased in the fibrotic heart (Fig. 1C). *In vitro*, miR-214 was upregulated by ISO in a dose- and time-dependent manner when compared to the control (Fig. 1C,D), suggesting that miR-214 may play a role in cardiac fibrosis.

### Downregulation of miR-214 attenuated ISO-induced proliferation and collagen synthesis of cardiac fibroblasts

**Figure 2:**
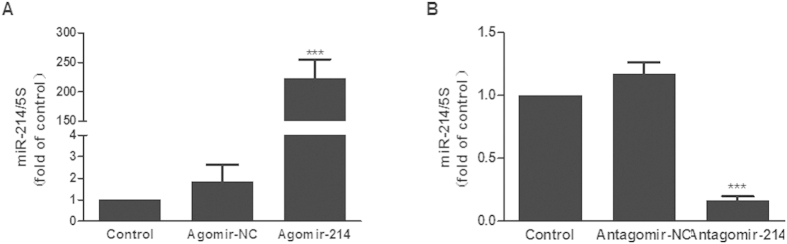
Overexpression or knockdown of miR-214 in CFs. (**A**) Transfection of agomir-214 (50 nM) in CFs significantly increased the expression of miR-214, as determined by quantitative real-time PCR. (**B**) Transfection of antagomir-214 (100 nM) in CFs significantly decreased the expression of miR-214 (n = 3, mean ± SEM, ^*^P < 0.05, ^**^P < 0.001, ^***^P < 0.001 *vs.* Control). NC: negative control.

To explore the role of miR-214 in cell proliferation and collagen synthesis, CFs were transfected with agomir-214 and antagomir-214 to overexpress and knock down miR-214, respectively. At 48 h post-transfection, miR-214 levels were measured by quantitative RT-PCR. Transfection with 50 nM agomir-214 augmented miR-214 expression, whereas transfection with 100 nM antagomir-214 reduced miR-214 levels significantly (Fig. 2A,B).

**Figure 3:**
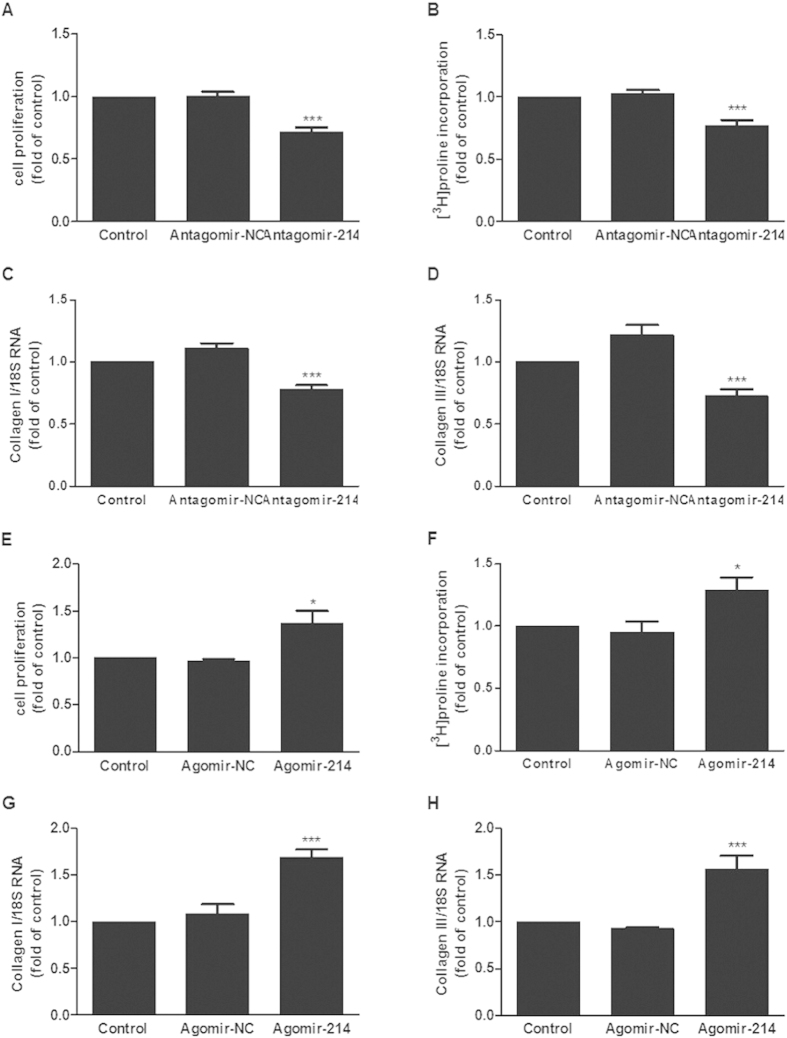
Downregulation of miR-214 attenuated the proliferation and collagen synthesis of CFs. Knockdown of miR-214 significantly decreased the proliferation (**A**), collagen synthesis rate (**B**) and the expression of collagens I and III (**C**,**D**) in CFs. Overexpression of miR-214 increased the proliferation (**E**), collagen synthesis rate (**F**) and the expression of collagens I and III (**G**,**H**) in CFs. (n = 3–4, mean ± SEM, *P < 0.05, ***P < 0.001 vs. NC).

The BrdU incorporation assay was applied to detect cell proliferation, and the ^3^H-Proline incorporation assay and collagen I and collagen III mRNA quantification assays were used to evaluate the collagen synthesis ability of CFs. Compared with negative control group, knockdown of miR-214 significantly decreased cell proliferation, collagen synthesis rate, collagen I and collagen III mRNA expression of cultured CFs (Fig. 3A–D), while overexpression of miR-214 increased cell proliferation and collagen synthesis (Fig. 3E,F).

**Figure 4:**
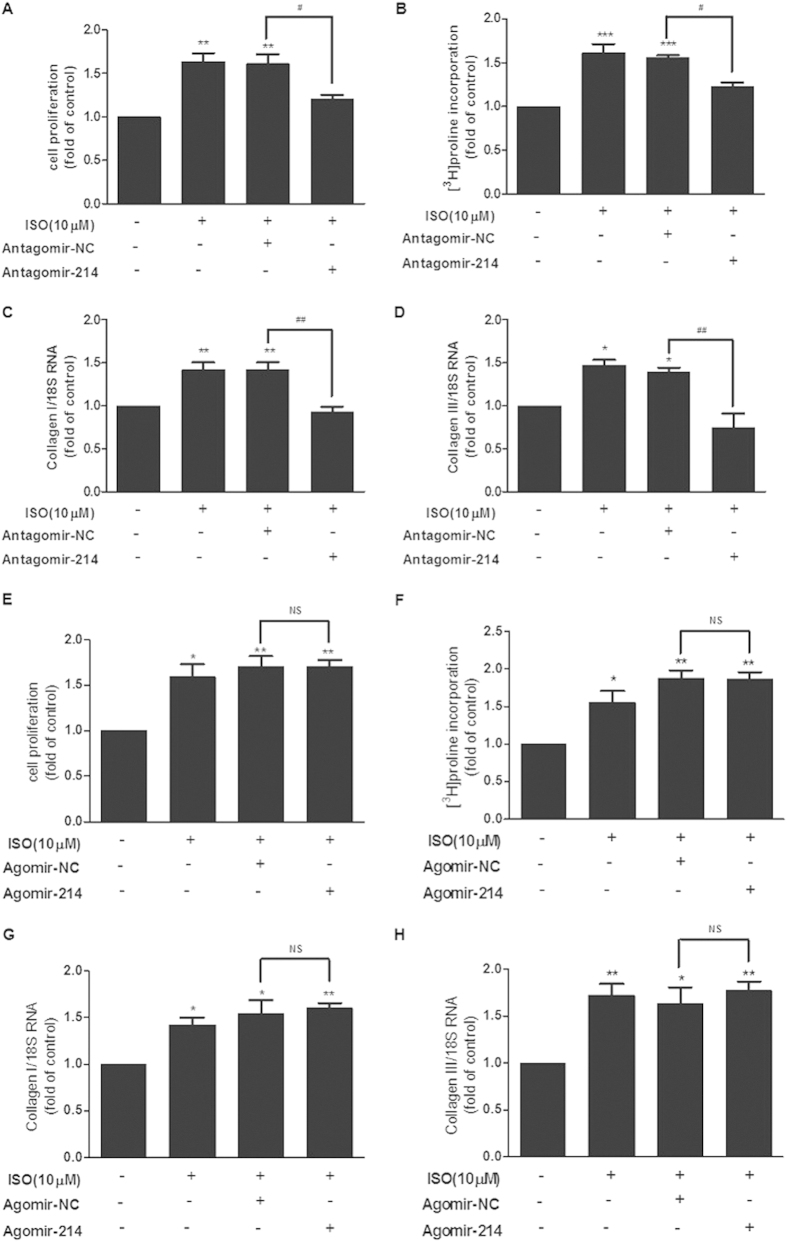
Downregulation of miR-214 attenuated ISO-induced proliferation and collagen synthesis of CFs. Knockdown of miR-214 significantly decreased ISO-induced proliferation (**A**), collagen synthesis rate (**B**) and the expression of collagens I and III (**C**,**D**) in CFs. Overexpression of miR-214 did not affect ISO-induced proliferation (**E**), collagen synthesis rate (**F**) or the expression of collagens I and III (**G**,**H**) in CFs. (n = 3–4, mean ± SEM, ^*^P < 0.05, ^**^P < 0.001, ^***^P < 0.001 *vs*. Control, ^#^P < 0.05 *vs*. ISO + NC, ^##^P < 0.01 *vs*. ISO + NC).

As shown in Fig. 4, ISO treatment increased cell proliferation, ^3^H-Proline incorporation and the mRNA levels of collagen I and collagen III in cultured CFs. In contrast, downregulation of miR-214 attenuated the increase in ISO-induced cell proliferation and collagen synthesis, demonstrating that miR-214 mediates ISO-induced cardiac fibroblast proliferation and collagen synthesis (Fig. 4A–D). However, miR-214 overexpression did not further accelerate the increase in cell proliferation and collagen synthesis when compared to ISO treatment (Fig. 4E–H).

### Mfn2 is the molecular target of miR-214

**Figure 5:**
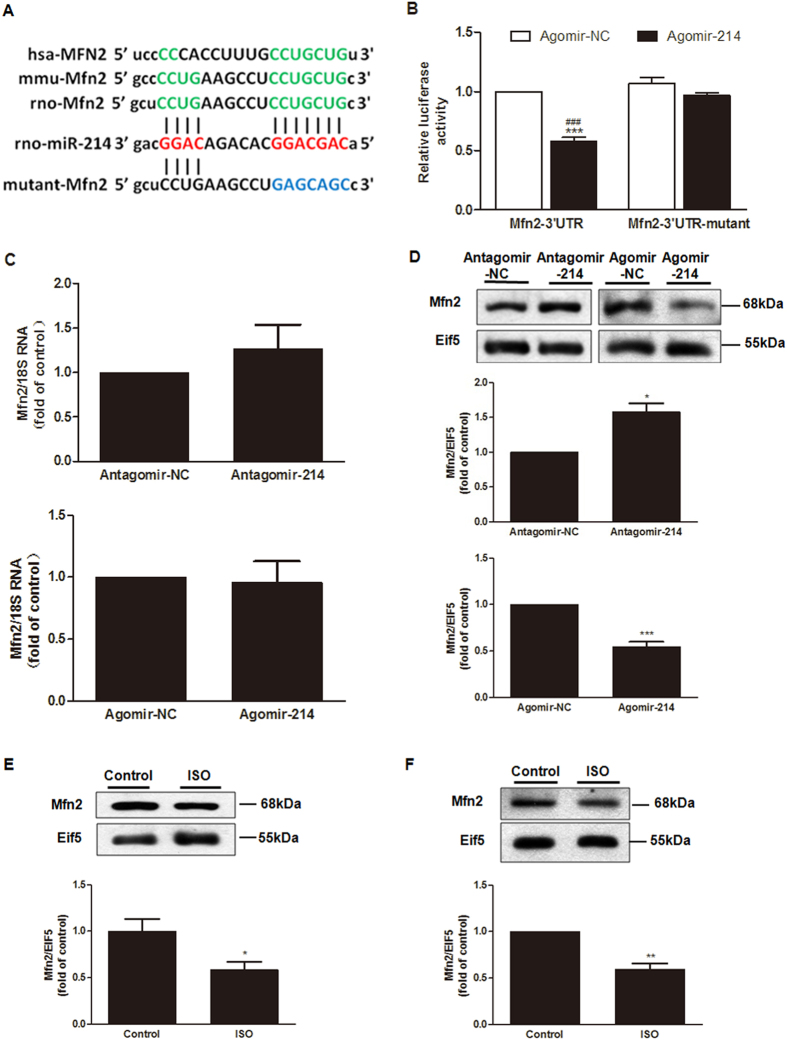
Mfn2 is the direct target gene of miR-214. (**A**) Conservation of the Mfn2 3′UTR binding site for miR-214 among different species and the mutated binding sites of Mfn2 3′UTR. (**B**) miR-214 targets the wild type but not the mutant 3′UTR of Mfn2. HEK293A cells were co-transfected with pGL_4_-Mfn2-3′UTR and agomir-214/NC or the pGL_4_-Mfn2–3′UTR mutant and agomir-214/NC for 48 h; then, luciferase activity was detected (n = 3, mean ± SEM, ^***^P < 0.001 *vs*. Mfn2 3′UTR + agomir-NC, ^###^P < 0.001 *vs*. Mfn2 3′UTR mutant + agomir-214). (**C**) MiR-214 did not affect Mfn2 mRNA expression, as determined by quantitative real-time PCR. (**D**) miR-214 negatively regulated the Mfn2 protein level in CFs, as determined by western blot analysis in CFs (n = 4, mean ± SEM, ^*^P < 0.05, ^***^P < 0.001 *vs*. NC). (**E**,**F**) Levels of Mfn2 protein were downregulated in ISO-induced fibrotic heart tissue and CFs (n = 4–6, mean ± SEM, ^*^P < 0.05, ^***^P < 0.001 *vs*. Control).

Because miRNAs negatively regulate gene expression at the post-transcriptional level by directly binding to the complementary sequences in the 3′UTRs of target genes, we searched several bioinformatics databases, including TargetScan (http://www. targetscan.org/), miRanda (http://www.microrna.org/microrna/home.do), PITA (http://genie.weizmann.ac.il/index.html) and miRWalk (http://www.umm. uniheidelberg.de/apps/zmf/mirwalk/), to identify the putative miR-214 target genes involved in the regulation of proliferation and collagen synthesis in CFs. The analysis predicted the sequence position 952–958 in the 3′UTRs of mitofusin2 (Mfn2) as a putative miR-214 binding site (Fig. 5A). This sequence is broadly conserved in human, mouse and rat models.

To identify whether Mfn2 is a direct target of miR-214 through this specific binding site, we constructed a wild-type luciferase reporter gene vector containing the 3′UTR of Mfn2 and a mutant vector with several mutations in the miR-214 binding site; we then co-transfected the reporter with agomir-214 in 293A cells. Compared with the control, the luciferase activity of the wild-type group was significantly lower, whereas the mutant group was not affected when miR-214 was overexpressed Fig. 5B.

In addition, real-time PCR and western blotting analysis were applied to investigate the effects of miR-214 on both Mfn2 mRNA and protein expression. The outcomes showed that the mRNA level of Mfn2 was not affected, but the protein level was correspondingly decreased or increased when miR-214 was overexpressed or downregulated (Fig. 5C,D), indicating that miR-214 negatively regulates Mfn2 expression by translational inhibition. Moreover, in accordance with the upregulation of miR-214, the endogenous Mfn2 protein level was decreased in both the ISO-induced cardiac fibrotic heart (*in vivo*) and in the CFs (*in vitro*), suggesting that Mfn2 levels are reduced by miR-214 during the pathogenesis of cardiac fibrosis.

### Inhibition of miR-214 reduces ISO-induced activation of ERK1/2–MAP kinase signalling

Previous studies have demonstrated that Mfn2 prevents vascular smooth muscle cell proliferation by inhibiting the Ras-Raf–ERK1/2 signalling pathway via direct binding with both Ras and Raf 20. ERK–MAP kinase signalling is a critical pathway that regulates CF proliferation and collagen synthesis^4^,^5^. Thus, we investigated the effect of miR-214 on ERK1/2 phosphorylation levels induced by ISO treatment.

**Figure 6:**
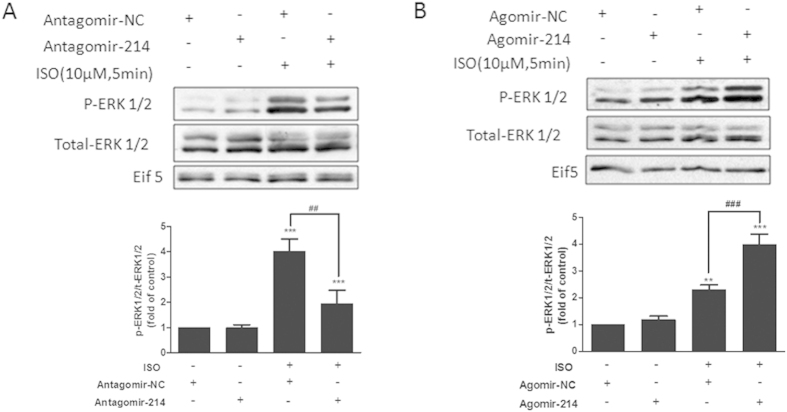
miR-214 positively regulates ISO-induced extracellular signal-regulated kinase 1/2 (ERK1/2) phosphorylation in CFs. (**A**) Downregulation of miR-214 inhibits ISO-induced ERK1/2 phosphorylation. CFs were transfected with antagomir-214, starved for 24 h, and then incubated with ISO (10 μM) for 5 min. (**B**) Overexpression of miR-214 promoted ISO-induced ERK1/2 phosphorylation. CFs were transfected with agomir-214, starved for 24 h, and then incubated with ISO (10 μM) for 5 min. The phosphorylation of ERK1/2 was analysed using western blotting (n = 4, mean ± SEM, ^*^P < 0.05, ^**^P < 0.001, ^***^P < 0.001 *vs.* Control).

As expected, downregulation of miR-214 significantly inhibited the increase of ERK1/2 phosphorylation induced by ISO treatment (Fig. 6A). In contrast, overexpression of miR-214 further promoted the increase in ERK1/2 phosphorylation (Fig. 6B).

## Discussion

MiRNAs widely participate in the pathogenesis of cardiac fibrosis^8^. In the present study, we found that miR-214 was significantly upregulated in a cardiac fibrosis model induced by ISO, a β-AR agonist. Downregulation of miR-214 attenuated ISO-induced cardiac fibroblast proliferation and collagen synthesis, indicating that the β-AR-induced cardiac fibrosis was partly mediated by a pathway that involved miR-214, which may provide new insight to elucidate the mechanism of β-AR-induced cardiac fibrosis.

Previous studies have revealed that miR-214 expression is significantly increased in the heart tissues of patients with ischemic heart disease, dilated cardiomyopathy and atherosclerosis^21^ as well as in the serum of chronic heart failure patients^22^. Similar results have also been obtained in animal models, where miR-214 is upregulated in both hypertrophic and failing heart tissues and cardiomyocytes^22^,^23^,^24^,^25^. Here, we found that miR-214 was significantly upregulated in β-AR-induced cardiac fibrotic heart tissues and fibroblasts. These studies indicate that miR-214 may serve as a biomarker or potential therapeutic target for cardiac remodelling.

In the cancer research field, miR-214 has been considered as a critical regulator of cell survival and proliferation. However, the conclusions thus far are controversial and depend on the tissues and target genes. In ovarian cancer and pancreatic cancer^26^,^27^, miR-214 has been found to act as an oncogene to promote cell proliferation and cisplatin resistance via the downregulation of PTEN and activation of the PI3K/AKT signalling pathway. In contrast, miR-214 serves as a tumour suppressor in cervical cancer, hepatocellular carcinoma and myeloma cells by inhibiting cell proliferation, invasion and migration^28^,^29^,^30^. In the present study, we demonstrated that miR-214 plays a positive role in cardiac fibroblast proliferation, indicating a tissue-selective effect of miR-214 on cell proliferation.

*In vivo* studies, miR-214 has been reported to participate in the process of negative cardiac remodelling because overexpression of miR-214 induces cardiac hypertrophy and cardiac dysfunction whereas inhibition of miR-214 can prevent cardiac hypertrophy, interstitial fibrosis, impairment of angiogenesis and cardiac dysfunction of heart failure^22^,^31^. In the present study, cardiac fibroblasts were transfected with agomir-214 and antagomir-214 referred to the preliminary experiments and other literatures^32^,^33^,^34^,^35^,^36^,^37^ and we found that the downregulation of miR-214 attenuated ISO-induced collagen synthesis in rat cardiac fibroblasts, which provides more evidence for the negative role of miR-214 in cardiac remodeling and helps to elucidate the possible mechanism. However, the miR-214 genetic deletion aggravated ischemia reperfusion injury-mediated cardiac fibrosis^24^. These contradictory results may be due to the different stimuli and compensatory mechanisms activated in the persistent absence of miR-214. Given that there are thousands of miRNAs working together in complicated networks *in vivo*, the genetic deletion of miR-214 may lead to compensation during development and thus may not reflect the real function. Moreover, the stimulus type may also affect the outcome.

Mfn2 is a protein that localizes to the outer membrane of the mitochondria; this protein plays an essential role in maintaining the morphology and function of the mitochondria^38^ and is a crucial regulator in controlling cell proliferation and tissue fibrosis^20^,^39^. Both *in vitro* and *in vivo* studies have demonstrated an inhibitory role of Mfn2 in cell proliferation for vascular smooth muscle cells (VSMCs)^20^. Furthermore, overexpression of Mfn2 can alleviate ECM deposition in the diabetic rat kidney^39^. In our study, Mfn2 was found to decrease in fibrotic rat heart tissues and fibroblasts and was negatively regulated by miR-214. This result indicates that miR-214-mediated cell proliferation and collagen synthesis occur at least partly via downregulation of Mfn2.

ERK1/2 MAPK signalling is a well-known modulator of CF growth and activation in the cardiovascular system. It has been demonstrated that β-AR agonists, such as ISO, can promote cardiac fibroblast proliferation and collagen synthesis by ERK1/2 MAPK activation^4^,^40^,^41^. Moreover, Mfn2 has been demonstrated to inhibit the Ras-Raf–ERK1/2 signalling pathway by directly binding with both Ras and Raf^20^. Therefore, we supposed that ERK1/2 MAPK signalling might be involved in miR-214-mediated CF activation. In line with our hypothesis, transfection of synthetic miR-214 antagonist led to a significant decrease in ISO-induced ERK1/2 MAPK activation, indicating that miR-214 could be a critical regulator of ERK1/2 MAPK activity in CFs.

In summary, our study reveals that ISO treatment increases the expression of miR-214 in both cultured neonatal fibroblasts and rat heart tissue. The upregulation of miR-214 can mediate ISO-induced proliferation and collagen synthesis in cardiac fibroblasts by regulating the target gene Mfn2 and its downstream ERK1/2 signalling pathway. Our research demonstrates that miR-214 is a new regulator of β-AR-mediated cardiac fibrosis and may serve as a novel therapeutic target in cardiac fibrosis.

## Methods

### Cardiac fibrosis model

The animal care and experimental procedures involved in this study were approved by the Institutional Animal Care and Use Committee of Peking University Health Science Center (LA2010-037) and adhered to the American Physiological Society’s “Guiding Principles in the Care and Use of Vertebrate Animals in Research and Training”.

A cardiac fibrosis model was induced in 180- to 200-g male Sprague–Dawley rats by subcutaneous injection of ISO (0.25 mg/kg) once a day for 7 consecutive days. A corresponding control group of animals received an equivalent volume of physiological saline. The rats were sacrificed by cervical dislocation after deep anaesthesia with 2% isoflurane (Baxter Healthcare Corporation, New Providence, NJ, USA) at the end of day 7. The walls of the ventricles were fixed with 4% (w/v) paraformaldehyde, dehydrated, and embedded in paraffin. Ventricle sections were stained with Sirius red to observe interstitial fibrosis. The collagen content of the myocardial tissue was determined by a hydroxyproline assay kit (Cat. No. A030-1; Jiancheng, Nanjing, China). The remaining samples were frozen in liquid nitrogen for later use.

### Cell culture

Neonatal cardiac fibroblasts were isolated from the hearts of 1- to 3-day-old Sprague-Dawley rats. Briefly, a central thoracotomy was performed after the neonatal rats were deeply anaesthetized with 1.0% isoflurane. The hearts were quickly excised and immediately embedded in freezing Hanks' solution. Cardiac fibroblasts were isolated from the rat hearts by enzymatic digestion with pancreatin and collagenase type II as described previously^42^, grown to 80% confluence and serum-starved for 24 h in serum-free medium before treatment with ISO (10 μmol/L) or transfection with agomir (50 nmol/L; RibioBio, Guangzhou, China) or antagomir (100 nmol/L; RibioBio) referred to the preliminary experiments and other literatures^32^,^33^,^34^,^35^,^36^,^37^.

### Real-Time PCR

Small RNAs were isolated using the miRcute miRNA isolation kit (Tiangen Biotech, Beijing, China). The miRcute microRNA first-strand cDNA synthesis and qPCR detection kits (Tiangen) were used for miRNA analysis. Quantitative PCR was performed using the ABI PRISM 7700 Sequence Detection System (Applied Biosystems, Invitrogen). Rat 5S ribosomal RNA was used as an internal control.

Total RNA was extracted using the TRIzol Reagent method (Invitrogen, Carlsbad, CA, USA). Relative quantification by real-time PCR was performed using SYBR Green-based detection of PCR products in real time with the ABI PRISM 7700 Sequence Detection System (Applied Biosystems, Invitrogen). Rat 18S ribosomal RNA was amplified as a reference standard. The reactions were prepared in triplicate and heated to 95 °C for 5 min, followed by 40 cycles of 94 °C for 30 s, 60 °C for 30 s, and 72 °C for 30 s. The primer sequences are shown in Table 1.

### Western Blot

Total protein was extracted in RIPA buffer (CxBio, Shanghai, China) supplemented with phenylmethanesulfonyl (PMSF, CxBio). The protein concentrations were measured using the BCA Protein Assay (Applygen, Beijing, China). The samples were separated on a 10% SDS-polyacrylamide gel and then transferred to a nitrocellulose membrane, which was blocked in 5% skim milk for 1 h and then incubated with primary antibodies at 4 °C overnight. After the washing steps, the membranes were incubated with horseradish peroxidase-labelled secondary antibodies for 1 h at room temperature. The bands were visualized using a chemiluminescence detection system, and the densitometric results were analysed with Image J software. EIF5 levels were used as internal controls for protein normalization.

Anti-Mfn2 and anti-EIF5 for western blotting were purchased from Santa Cruz Biotechnology (CA, USA), and anti-phospho-ERK1/2 and anti-total-ERK1/2 antibodies were purchased from Cell Signalling Technology (Beverly, MA, USA).

### Dual-Luciferase Reporter Assay

The sequence of the miR-214 predicted target region was synthesized from the 3′UTR of rat Mfn2 and cloned into the dual luciferase reporter vector pmiRGLO. A control construct with mutations incorporated in the miR-214 seed region was generated in a similar manner. The sequences of these constructs were verified. HEK293 cells were transfected with 200 ng of plasmid DNA (wild-type PGL4-Mfn2-3′UTR/mutant PGL4-Mfn2-3′UTR which were commercially constructed using GeneChem (Shanghai GeneChem Co., Ltd.)) and agomir (50 nmol/L; RibioBio) using Lipofectamine 2000 according to the manufacturer’s instructions (Invitrogen). The luciferase activity was measured by the Dual-Luciferase Reporter Assay System (Promega, Madison, WI, USA).

### ^3^H-Proline Incorporation Assay

The collagen synthesis was determined by the quantification of ^3^H-Proline incorporation. In brief, CFs were plated on 24-well culture dishes at a density of 5 × 10^4^ cells/well and treated with or without various reagents, as mentioned above. The cells were labelled with ^3^H-Proline (1 μCi) for the last 24 h, fixed in 10% trichloroacetic acid, and collected in 0.01 M NaOH and 1 g/L SDS. Aliquots for each treatment were counted in a liquid scintillation counter using 3 ml of scintillation fluid.

### BrdU Incorporation Assay

Proliferation was assessed by the quantification of 5-bromo-2′-deoxyuridine (BrdU) incorporation. In brief, CFs were plated on 96-well culture dishes at a density of 1 × 10^4^ cells/well and treated with or without various reagents, as mentioned above. Then, BrdU (10 μmol/L) was added to the medium, and the cells were incubated for another 6 h. Subsequently, the cells were fixed, and BrdU incorporation was determined with a Cell Proliferation ELISA Kit (Roche Diagnostics, Mannheim, Germany) according to the manufacturer’s instructions.

### Statistical Analysis

All of the experiments were performed at least three times. The data are expressed as means ± SEMs and were analysed by ANOVA and a post-hoc Tukey’s analysis or by a t-test as appropriate. A p value of 0.05 or less was considered significant.

## Additional Information

**How to cite this article**: Sun, M. *et al.* MicroRNA-214 Mediates Isoproterenol-induced Proliferation and Collagen Synthesis in Cardiac Fibroblasts. *Sci. Rep.* **5**, 18351; doi: 10.1038/srep18351 (2015).

## Figures and Tables

**Table 1:** List of primers used in qRT-PCR.

	Sense primers (5′–3′)	Antisense primers (5′–3′)
5s	GTCTACGGCCATACCACCCTGAAC	Provided by miScript SYBR Green PCR Kit(Tiangen Biotech, Beijing, China)
miR-214	ACAGCAGGCACAGACAGGCAG	
Mfn2	ATGATAGACGGCTTGAA	CGACTCCCTCTTTGTGA
18s rRNA	TACCACATCCAAGGAAGGCAGCA	TGGAATTACCGCGGCTGCTGGCA
Collagen I	ATCAGCCCAAACCCCAAGGAGA	CGCAGGAAGGTCAGCTGGATAG
Collagen III	TGATGGGATCCAATGAGGGAGA	GAGTCTCATGGCCTTGCGTGTTT
